# The prognosis, chemotherapy and immunotherapy efficacy of the SUMOylation pathway signature and the role of UBA2 in lung adenocarcinoma

**DOI:** 10.18632/aging.205594

**Published:** 2024-02-23

**Authors:** Liying Yu, Na Lin, Yan Ye, Haohan Zhuang, Shumei Zou, Yingfang Song, Xiaoli Chen, Qingshui Wang

**Affiliations:** 1 Central Laboratory, The Second Affiliated Hospital of Fujian Medical University, Quanzhou, Fujian 362000, China; 2 Department of Pathology, The Second Affiliated Hospital of Fujian Medical University, Quanzhou, Fujian 362000, China; 3 Jiangxi Health Commission Key Laboratory of Leukemia, The Affiliated Ganzhou Hospital of Nanchang University, Ganzhou, Jiangxi 341000, China; 4 Laboratory Animal Center, The Second Affiliated Hospital of Fujian Medical University, Quanzhou, Fujian 362000, China; 5 900 Hospital of The Joint Logistics Team, Fuzhou, Fujian 350001, China; 6 Department of Pulmonary and Critical Care Medicine, Fuzong Clinical College of Fujian Medical University, Fuzhou, Fujian 350001, China; 7 Dongfang Hospital of Xiamen University, School of Medicine, Xiamen University, Xiamen, Fujian 361000, China; 8 Fujian-Macao Science and Technology Cooperation Base of Traditional Chinese Medicine-Oriented Chronic Disease Prevention and Treatment, Innovation and Transformation Center, Fujian University of Traditional Chinese Medicine, Fuzhou, Fujian 350001, China

**Keywords:** LUAD, SUMOylation, prognosis, UBA2, treatment

## Abstract

Lung adenocarcinoma (LUAD) is one of the most common malignant tumors worldwide. Small Ubiquitin-like Modifier (SUMO)-ylation plays a crucial role in tumorigenesis. However, the SUMOylation pathway landscape and its clinical implications in LUAD remain unclear. Here, we analyzed genes involved in the SUMOylation pathway in LUAD and constructed a SUMOylation pathway signature (SUMOPS) using the LASSO-Cox regression model, validated in independent cohorts. Our analysis revealed significant dysregulation of SUMOylation-related genes in LUAD, comprising of favorable or unfavorable prognostic factors. The SUMOPS model was associated with established molecular and histological subtypes of LUAD, highlighting its clinical relevance. The SUMOPS stratified LUAD patients into SUMOPS-high and SUMOPS-low subtypes with distinct survival outcomes and adjuvant chemotherapy responses. The SUMOPS-low subtype showed favorable responses to adjuvant chemotherapy. The correlations between SUMOPS scores and immune cell infiltration suggested that patients with the SUMOPS-high subtype exhibited favorable immune profiles for immune checkpoint inhibitor (ICI) treatment. Additionally, we identified UBA2 as a key SUMOylation-related gene with an increased expression and a poor prognosis in LUAD. Cell function experiment confirmed the role of UBA2 in promoting LUAD cell proliferation, invasion, and migration. These findings provide valuable insights into the SUMOylation pathway and its prognostic implications in LUAD, paving the way for personalized treatment strategies and the development of novel therapeutic targets.

## INTRODUCTION

Lung cancer is the leading cause of cancer-related deaths, contributing to 18% of total cancer mortalities according to the 2020 global cancer statistics [1]. Lung cancer is divided into two main categories: small cell lung cancer (SCLC) and non-small cell lung cancer (NSCLC), with NSCLC representing around 85% of all lung cancer cases [2, 3]. Based on distinct pathological features, NSCLC is further classified into subtypes of lung adenocarcinoma (LUAD) and lung squamous cell carcinoma (LUSC), among which LUAD is the most prevalent [4]. Despite notable progress in clinical treatments such as targeted therapies utilizing tyrosine kinase inhibitors for LUAD, the 5-year survival rate remains moderately low [5, 6]. Consequently, there is an urgent requirement for more effective and dependable prognostic evaluation indicators to accurately identify patients who will benefit from specific drug treatment regimens [7, 8].

SUMOylation is an important protein modification process that regulates many biological processes within cells. SUMO protein can covalently attach to other proteins and alter protein function, stability, and subcellular localization [9]. SUMOylation involves three key members: SUMO-activating enzyme (E1), SUMO-conjugating enzyme (E2), and SUMO ligase (E3) [10]. The SUMO-activating enzyme is responsible for activating the SUMO protein. The SUMO-activating enzyme is encoded by two genes: Sentrin/SUMO-specific activating enzyme 1 (SAE1) and SAE2 (also known as UBA2). SAE1 and SAE2 form a complex that conjugates ATP to the C-terminus of the SUMO protein, generating an activated SUMO intermediate. The SUMO-conjugating enzyme transfers the activated SUMO from E1 to the target protein. In humans, the SUMO-conjugating enzyme is primarily encoded by a gene, ubiquitin-conjugating enzyme 9 (Ubc9). Ubc9 forms a thioester bond with the activated SUMO intermediate and transfers the SUMO moiety onto the target protein. The SUMO ligase is responsible for regulating the location and effects of SUMOylation. Multiple E3 ligases participate in SUMOylation in humans. Each E3 ligase has specific substrate selectivity and forms a complex with the SUMO-conjugating enzyme and the substrate to transfer SUMO onto the target protein. Research has shown that SUMOylation plays a crucial role in tumor development, such as influencing tumor cell proliferation, apoptosis, cell cycle regulation, and DNA repair [11–13].

In the study, we performed a comprehensive analysis of SUMOylation pathway-related genes in LUAD and developed a SUMOylation pathway signature (SUMOPS) with robust prognostic value. The SUMOPS model represents a promising avenue for improving LUAD patient management by providing valuable prognostic information and guiding the selection of appropriate therapeutic interventions.

## METHODS

### Clinical data collection and extraction

The publicly available TCGA-LUAD and 10 GEO datasets [14–22] were utilized in this study (Table 1). The TCGA-LUAD cohort comprised 515 LUAD and 59 adjacent non-tumor samples. Its corresponding transcriptomic and clinical data were downloaded from TCGA (https://portal.gdc.cancer.gov/). The GEO datasets were downloaded from the GEO database (https://www.ncbi.nlm.nih.gov/geo/). Among these datasets, six have clinical survival information (TCGA-LUAD, GSE11969, GSE13213, GSE26939, GSE68465, and GSE72094), and two hold parts of patients receiving adjuvant chemotherapy (TCGA-LUAD and GSE68465). Two datasets have molecular subtype information, including GSE26939 and GSE58772. Within the GSE26939 cohort, LUAD patients were classified into three molecular subtypes: bronchioalveolar carcinoma (bronchioid, *n* = 47), squamous cell carcinoma (squamoid, *n* = 29), and large-cell carcinoma (magnoid, *n* = 40) [19]. The GSE58772 cohort classified LUAD patients into five molecular subtypes: lepidic (*n* = 10), acinar (*n* = 10), papillary (*n* = 9), micropapillary (*n* = 9), and solid predominant adenocarcinoma (*n* = 10) [22].

**Table 1:** Information on lung adenocarcinoma cohorts from public datasets used in this study.

**Source**	**Dataset number**	**No. of LUAD tissues**	**No. of adjacent non-tumor tissues**	**No. of patients who received adjuvant chemotherapy**	**With clinical survival information or not**	**With molecular subtype or not**	**References**
TCGA	TCGA-LUAD	515	59	159	Yes	No	–
GEO	GSE10072	58	49	–	No	No	12
	GSE18842	46	45	–	No	No	13
	GSE33479	13	27	–	No	No	14
	GSE33532	80	20	–	No	No	–
	GSE11969	148	–	–	Yes	No	15
	GSE13213	117	–	–	Yes	No	16
	GSE26939	116	–	–	Yes	Yes	17
	GSE68465	443	–	85	Yes	No	18
	GSE72094	442	–	–	Yes	No	19
	GSE58772	58	–	–	No	Yes	20

### Construction and validation of the SUMOPS prognostic risk model

Human Gene Set (GO-BP protein SUMOylating) was obtained from Molecular Signatures Database (MSigDB, https://www.gsea-msigdb.org/gsea/msigdb). LASSO (Least Absolute Shrinkage and Selection Operator) is a technique commonly used to fit sparse regression models with high-dimensional predictors. It presents an alternative method for variable selection by applying the L1 penalty, which allows coefficient estimates to be forced to zero. By employing the logistic regression model with the LASSO penalty, variable shrinkage and selection can be effectively achieved. In this study, the LASSO regression analysis is conducted using the ‘glmnet’ package in R. This package specifically offers functions and tools tailored for implementing LASSO regression. Nine SUMOylation related genes were sleeted by LASSO and used to construct a risk score model. Accordingly, we classified LUAD samples into SUMOPS-low and SUMOPS-high subtypes using the media SUMOPS score as a cutoff. Differences in overall survival (OS) between these two subtypes were analyzed in both the training set (TCGA-LUAD) and five testing sets (GSE11969, GSE13213, GSE26939, GSE68465, and GSE72094) using Kaplan-Meier (KM) plots.

### Meta-analysis

To assess the prognostic value of the developed SUMOPS model, a meta-analysis was conducted using the Onlinemeta tool (https://smuonco.shinyapps.io/Onlinemeta/).

### Drug sensitivity evaluation

To explore potential molecular compounds suitable for targeted therapy, we analyzed the drug sensitivity of the SUMOPS genes based on the Cancer Therapeutics Response Portal (CTRP) database (https://portals.broadinstitute.org/ctrp.v2.1/). This database provided valuable insights into the sensitivity of the genes toward various drugs.

### Genetic alteration and functional enrichment analyses

We obtained the somatic mutation data of LUAD from TCGA on the UCSC XENA website (http://xena.ucsc.edu/) [23]. The expression difference of the SUMOPS genes between different pathway activity groups (activation and inhibition) was assessed using Pathway Activity analysis on the Gene Set Cancer Analysis (GSCA) website (http://bioinfo.life.hust.edu.cn/GSCA/#/) [24]. The activity groups were defined based on pathway scores. Gene set variation analysis (GSVA) was employed to assess the pathway differences between the SUMOPS-low and SUMOPS-high subtypes. This analysis involved estimating variations in pathway activities using hallmark gene sets from MSigDB.

### Analysis of the prognostic value of SUMOPS after receiving ICIs therapy

The Tumor Immune Dysfunction and Exclusion (TIDE) score table, including the TIDE score, exclusion immune rejection, and dysfunction, was obtained from the TIDE website (http://tide.dfci.harvard.edu) [25, 26] based on TCGA-BLCA transcriptome data of LUAD patients.

### Tumor immune microenvironment analysis

The CIBERSORT algorithm was used to estimate immune cell infiltration. Based on the gene expression matrix of the TCGA-LUAD cohort, we estimated the infiltration of 22 different types of immune cells in LUAD patients. Furthermore, we compared relative abundance differences in these 22 immune cell types between the SUMOPS-low and SUMOPS-high subgroups.

### Cell culture and transfection

A549 and H1299 cell lines were obtained from American Type Culture Collection (ATCC) (Manassas, VA, USA) and cultured in RPMI 1640 medium appending with 10% fetal calf serum (FBS, Gibco, Carlsbad, CA, USA) and 1% double resistant (100 U/mL penicillin and 0.1 mg/mL streptomycin) under a humidified atmosphere of 5% CO_2_ at 37°C. To generate stable UBA2 knockdown A549 and H1299, two UBA2 shRNA sequences were cloned into the PLVX-shRNA plasmid, designated as UBA2-shRNA1 and UBA2-shRNA2. The UBA2-shRNA1 and UBA2-shRNA2 were packaged into lentiviruses. Cells were infected with the UBA2-shRNA1 and UBA2-shRNA2 lentivirus, respectively. Finally, the cell line was obtained after puromycin screening.

### Invasion assays

The invasion capacity of A549 and H1299 cells was detected by transwell assay. Cells (6 × 10^6^) in 300 μL serum-free medium were added to 24-well plates with an invasion chamber. The underlying chamber was added 500 μL of complete medium and placed at 37°C incubation of 5% CO_2_ for 48 h. The invaded cells passing through the membrane were fixed with methanol and stained with 500 μL 0.1% crystal violet for 10–20 min. Cells on the overhead chamber surface with cotton swabs to wipe off, the quantity of making inroads on tumor cells were photographed stochastically at 6 spotting areas.

### Migration assays

The migration capacity of A549 and H1299 cells was evaluated by wound healing assay. Cells (3 × 10^6^) were cultured in 6-well plates and aggregated to 85–90%. Before scratched, 10% PBS was used to wash the dislodged cellular debris away at least 3 times and left a little liquid to prevent cell death. And then a 10 μL spear made use of scratching wounds, and 10% PBS was used to wash the dislodged cellular debris away. In addition, serum-free medium was added to the plates. The cells were cultured in a place at 37°C incubation of 5% CO_2_ for 24 h and filmed microscopically again. Finally, ImageJ was used to figure up the migration capacity of A549 and H1299 cells.

### Cell proliferation assay

Adding 200 μL diluted cells into 96-well plates with 6 repeat wells in each cell group. After cell adherence, we take out the 96-well plate of 0 h, 24 h, 48 h, and 72 h. Next, 100 μL medium was sucked out for each well and 10 μL CCK-8 solution was added and cultured in a place at 37°C incubation of 5% CO_2_ for 1 h, and then the absorbance was measured at 450 nm with a microplate reader.

### Statistical analysis

Groups were compared using the Student’s *t*-test, and the results were presented as mean ± standard deviation (SD). Two-way analysis of variance (ANOVA) was employed to analyze the CCK8 assay. Statistical significance was defined as *p* < 0.05 for all tests conducted.

### Availability of data and materials

The datasets analyzed for this study can be found in the TCGA-LUAD (https://portal.gdc.cancer.gov) and GEO (https://www.ncbi.nlm.nih.gov/geo/).

## RESULTS

### The landscape of SUMOylation pathway-related genes in LUAD

**Figure 1:**
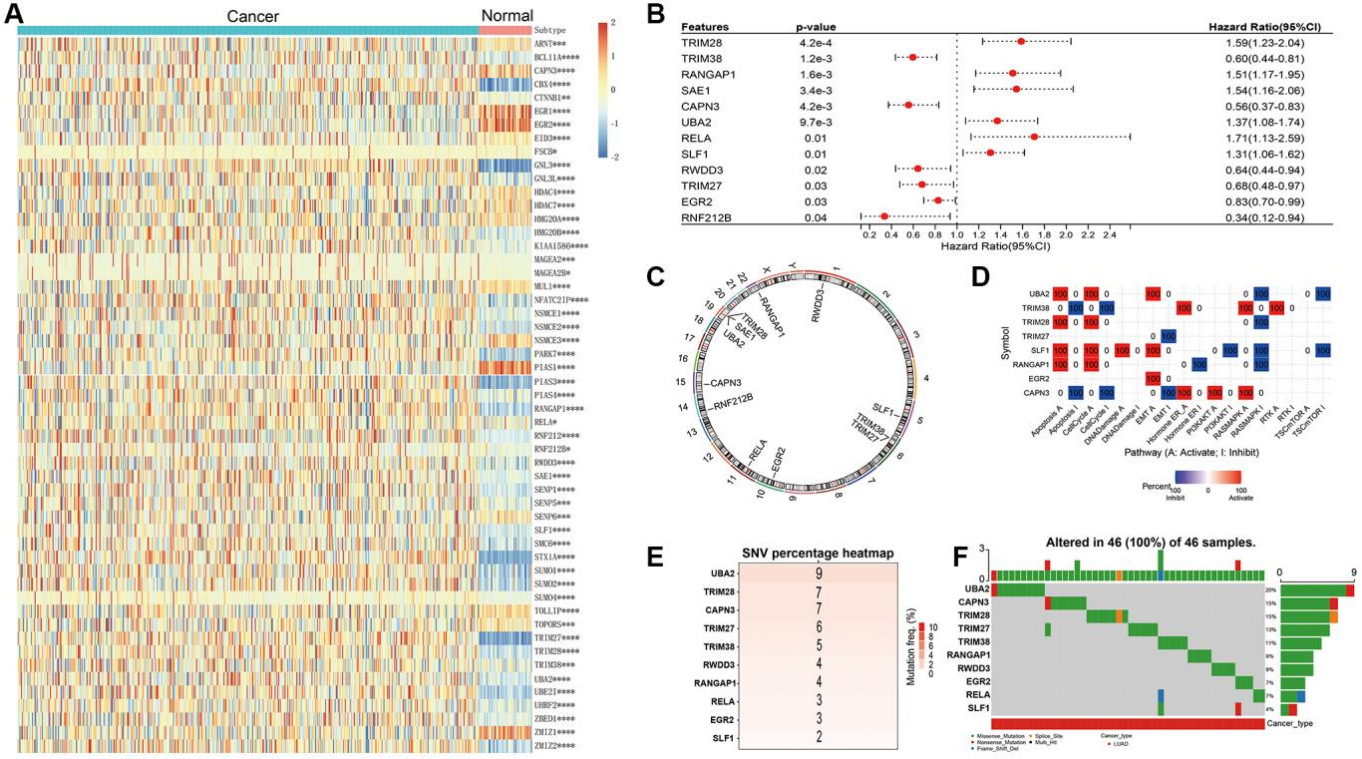
**Landscape of genetic variation and correlation of SUMOylation pathway-related genes in LUAD.** (**A**) The heat map demonstrates the differential expression of SUMOylation pathway genes between LUAD and adjacent non-tumor tissues. (**B**) Univariate survival analysis results of SUMOylation pathway genes in LUAD patients. (**C**) The chromosomal locations of the 12 SUMOylation pathway genes were determined. (**D**) Pathway analysis of SUMOylation pathway genes in LUAD. (**E**) The number of patients with mutations in SUMOylation pathway genes using TCGA-LUAD cohort. (**F**) Oncoplots showing the mutation landscape of SUMOylation pathway genes in LUAD patients from TCGA-LUAD cohort. ^*^p < 0.05; ^**^p < 0.01, ^***^p < 0.001, ^****^p < 0.0001.

SUMOylation-related gene analysis revealed that 38 and 15 out of 68 total SUMOylation-related genes were significantly upregulated and downregulated in LUAD tissues compared with normal tissues, respectively (Figure 1A). Survival analysis further demonstrated that elevated expression of Tripartite motif-containing 28 (TRIM28), Ran GTPase-activating protein 1 (RANGAP1), SUMO-activating enzyme subunit 1 (SAE1), Ubiquitin-like modifier-activating enzyme 2 (UBA2), v-rel avian reticuloendotheliosis viral oncogene homolog A (RELA), and SMC5-SMC6 Complex Localization Factor 1 (SLF1) were associated with unfavorable prognosis in LUAD patients. Conversely, higher expression of Tripartite motif-containing 38 (TRIM38), Calpain 3 (CAPN3), Peptidylprolyl isomerase domain and WD repeat-containing protein 3 (PWDD3), Tripartite motif-containing 27 (TRIM27), Early growth response 2 (EGR2), and Ring finger protein 212B (RNF212B) was linked to favorable prognosis in LUAD patients (Figure 1B). Further examination revealed that these 12 genes are all located on autosomes (Figure 1C). Functional analysis results indicated that UBA2, TRIM28, SLF1 and RANGAP1 exhibited similar functions, with positive correlations to Apoptosis, Cell cycle, yet negative correlations to Rat Sarcoma/Mitogen-Activated Protein Kinase (RAS/MAPK). TRIM38 and CAPN3 displayed positive correlations with Hormone Estrogen Receptor (ER) and RAS/MAPK, but negative correlations with Apoptosis and Cell cycle (Figure 1D). Gene mutation analysis indicated that UBA2, TRIM28, and CAPN3 were the top three genes with the most mutation frequency, in decreasing order (Figure 1E). Notably, the mutations of the 10 genes were detected in 46 LUAD patients. Each patient had mutations detected in only one specific gene of them, except for one patient who had concurrent mutations in CAPN3 and TRIM27, one patient with mutations simultaneously detected in TRIM38, RELA, and SLF1, and one patient with mutations in both SLF1 and EGR2 (Figure 1F). This suggests that TRIM28, TRIM38, RANGAP1, SAE1, CAPN3, UBA2, RELA, SLF1, RWDD3, TRIM27, EGR2, and RNF212B exhibit a relatively low mutation rate in LUAD.

### Construction of a prognostic SUMOylation pathway signature

**Figure 2:**
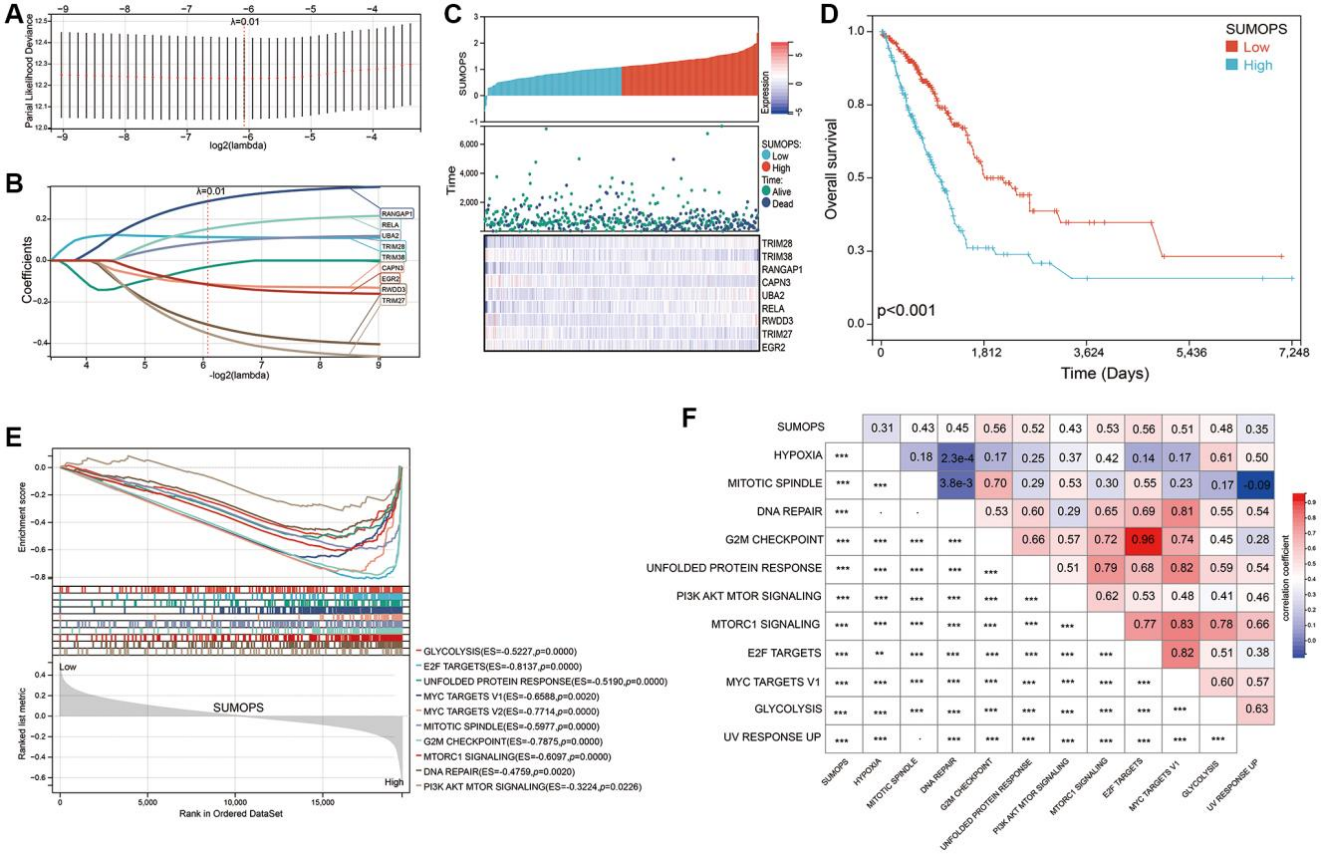
**Construction of a SUMOPS to predict the prognosis of LUAD patients.** (**A**) LASSO coefficient profile illustrating the relationship between overall survival and partial likelihood deviation. (**B**) Distribution of LASSO coefficients for the SUMOPS genes, indicating their respective contributions to the model. (**C**) Distribution of SUMOPS expression, survival status, and SUMOPS gene expression in the TCGA-LUAD dataset. (**D**) Prognostic analysis investigating the role of SUMOPS in predicting outcomes in the TCGA-LUAD cohort. (**E**) ssGSEA analysis of SUMOPS-low and SUMOPS-high subtypes. (**F**) Correlation analysis using Spearman’s rank correlation to examine the associations between SUMOPS and known gene signatures. ^−^*p* > 0.05; ^**^p < 0.01, ^***^p < 0.001.

The utilization of the LASSO-Cox regression model, coupled with cross-validation, allowed us to pinpoint the most influential SUMOylation-related genes that significantly affect the prognosis of patients with LUAD. As a result, we identified 9 specific genes with the best predictive performance in terms of minimizing the partial likelihood of deviance (Figure 2A). The LASSO coefficient profile revealed the relationships between the magnitude of lambda (λ) and the number of gene combinations (Figure 2B). Consequently, 9 genes related to the SUMOylation pathway were selected and utilized to construct a SUMOylation pathway signature (SUMOPS) to accurately evaluate LUAD prognosis. The SUMOPS score = (0.11 × the expression of TRIM28) + (−0.03 × the expression of TRIM38) + (0.28 × the expression of RANGAP1) + (−0.11 × the expression of CAPN3) + (0.08 × the expression of UBA2) + (0.15 × the expression of RELA) + (−0.31 × the expression of RWDD3) + (−0.35 × the expression of TRIM27) + (−0.11 × the expression of EGR2). The evaluated SUMOPS scores hinted decreased survival times, poorer outcomes, and changed gene expression patterns (Figure 2C). We also observed patients with the SUMOPS-low subtype exhibited markedly improved survival compared to patients with the SUMOPS-high subtype (Figure 2D). The hallmark enrichment analysis demonstrated significant enrichments of cell proliferation-related gene sets in the SUMOPS-high subtype, including the Glycolysis, E2F targets, Unfolded protein response, MYC targets V1, MYC targets V2, Mitotic spindle, G2M checkpoint, MTORC1 signaling, DNA repair and PI3K/AKT/MTOR signaling (Figure 2E). Correlation analysis results showed that the SUMOPS value was significantly positively correlated with these pathways (Figure 2F). These results imply these pathways might have considerable therapeutic potential for LUAD patients with the SUMOPS-high subtype.

Next, we explored the correlation between SUMOPS and clinical subtypes of lung cancer. For male and female patients, as well as younger (age ≤60) and older (age >60) patients, those classified as the SUMOPS-low subtype exhibited significantly better prognosis compared to those classified as the SUMOPS-high subtype (Supplementary Figure 1). Clinical staging is known to influence prognosis. Specifically, within the early-stage subgroup, patients in the SUMOPS-high subtype had shorter overall survival (OS) compared to patients in the SUMOPS-low subtype (Supplementary Figure 1). Similarly, in the late-stage subgroup, patients with the SUMOPS-high subtype had worse OS than those in the SUMOPS-low subtype. Also, for subgroups categorized by T-stage (T = 1&2, T = 3&4), M-stage (M = 0, M = 1&X), and N-stage (*N* = 0, *N* = 1,2,3&X), patients with the SUMOPS-high subtype experienced shorter OS. Considering the significant impact of tumor recurrence on the prognosis of LUAD patients, we conducted further analysis stratifying the OS of patients in recurrent and non-recurrent subgroups. The results revealed that the SUMOPS can accurately stratify the OS between the recurrent and non-recurrent subgroups (Supplementary Figure 1). These results highlight the robust predictive ability of SUMOPS in forecasting the prognosis of LUAD patients.

### Validation of the prognostic SUMOylation pathway signature

**Figure 3:**
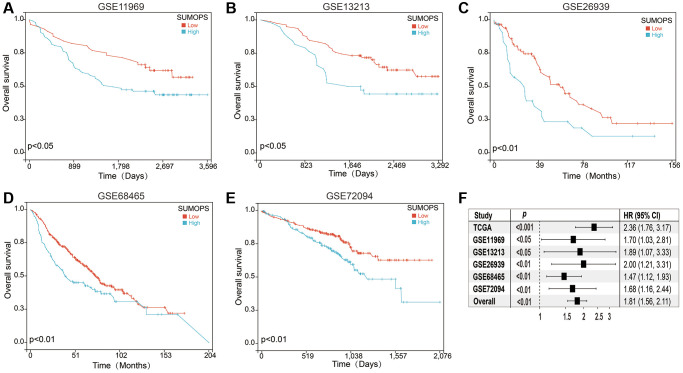
**Validation of the SUMOPS model to predict the prognosis of LUAD patients.** (**A**–**E**) Prognostic analysis investigating the role of SUMOPS in predicting outcomes within the (**A**) GSE11969, (**B**) GSE13213, (**C**) GSE26939, (**D**) GSE68465, and (**E**) GSE72094 cohort. (**F**) The meta-analysis indicated that the LUAD patients with high SUMOPS suffered poorer overall survival.

To thoroughly evaluate and validate the prognostic efficacy of the SUMOPS model, we extended our analysis to encompass five additional independent cohorts (Figure 3A–3E). By implementing the SUMOPS model in the GSE11969, GSE13213, GSE26939, GSE68465, and GSE72094 cohorts, we found that patients classified as the SUMOPS-high subtype exhibited significantly inferior overall survival outcomes compared to those categorized as SUMOPS-low subtype. This uniformity across multiple cohorts strengthens the validity and generalizability of the SUMOPS model in predicting prognosis in LUAD patients. Additionally, we conducted an extensive meta-analysis incorporating both the five GEO cohorts and the TCGA cohort (Figure 3F). The outcomes of this analysis further reaffirmed the predictive power of SUMOPS, underscoring its role as a valuable risk stratifier for LUAD.

### Analysis of molecular characteristics in SUMOPS-low and SUMOPS-high subtypes

**Figure 4:**
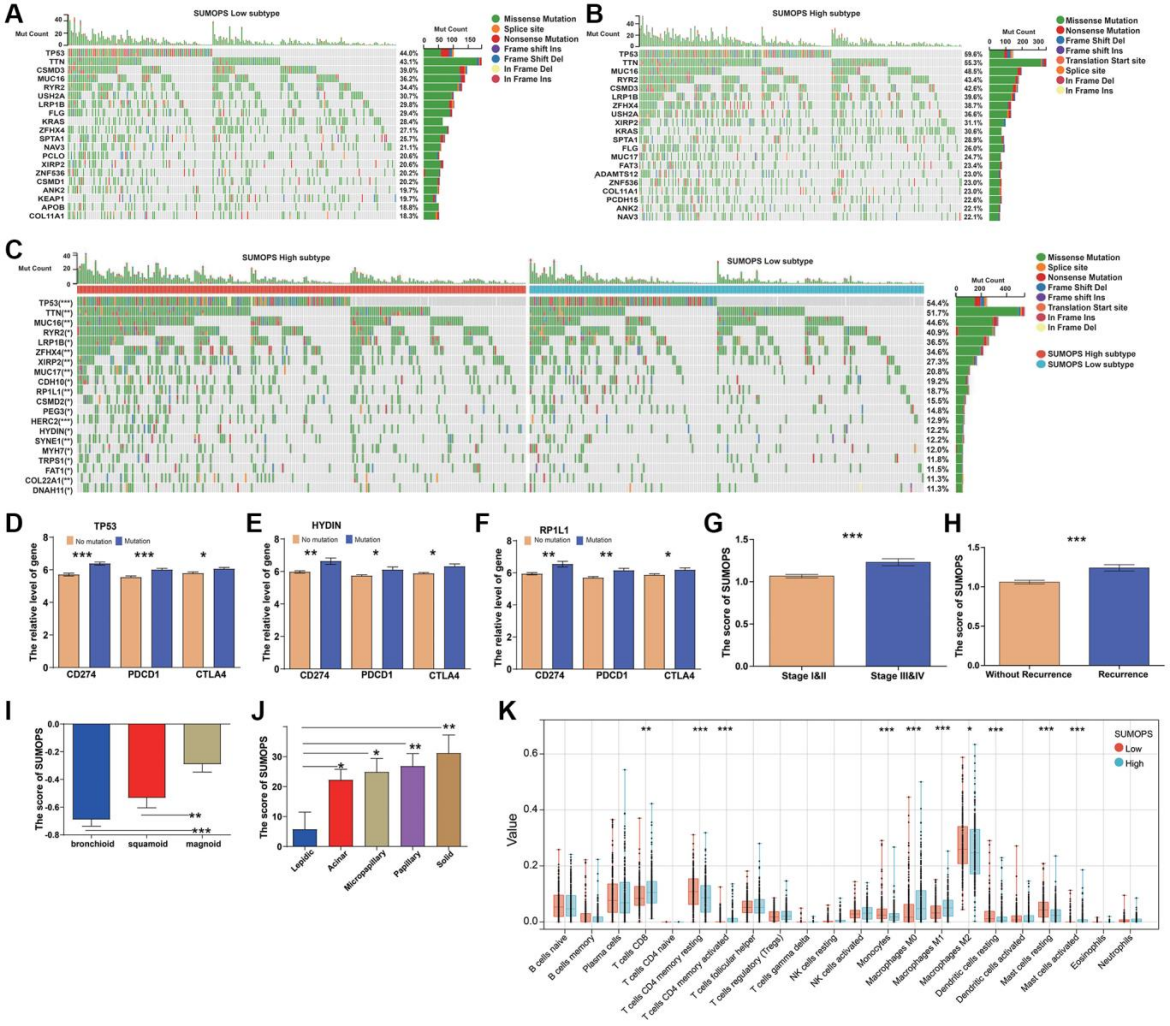
**Relationship between the SUMOPS and genomic alterations as well as molecular subtypes in LUAD.** (**A**, **B**) Oncoplots showing landscapes of genomic alterations in (**A**) SUMOPS-low and (**B**) SUMOPS-high subtypes. (**C**) Top 20 SUMOPS-related genes with the highest mutation frequency based on TCGA-LUAD cohort. (**D**) TP53, (**E**) HYDIN and (**F**) RP1L1 mutations distinctly facilitated expression of immune checkpoints (CTLA4, CD274, and PDCD1). (**G**) The score of SUMOPS at different stages. (**H**) The score of SUMOPS in recurrence and non-recurrence LUAD patients. (**I**) The score of SUMOPS in different molecular subtypes based on the GSE26939 cohort. (**J**) The score of SUMOPS in different molecular subtypes based on the GSE58772 cohort. (**K**) Box plots illustrating the relationships between SUMOPS subtypes and the infiltration of immune cells. ^*^p < 0.05; ^**^p < 0.01, ^***^p < 0.001.

We further conducted a genomic analysis of the variations in the SUMOPS-low and SUMOPS-high subtypes. In the SUMOPS-low subtype, the top 20 genes with the most mutation frequency included TP53 (44%) with the highest rate, followed by TTN (43.1%), CSMD3 (39%), MUC16 (36.2%), RYR2 (34.4%), USH2A (30.7%), LRP1B (29.8%), FLG (29.4%), KRAS (28.4%), ZFHX4 (27.1%), SPTA1 (25.7%), NAV3 (21.1%), PCLO (20.6%), XIRP2 (20.6%), ZNF536 (20.2%), CSMD1 (20.2%), ANK2 (19.7%), KEAP1 (19.7%), APOB (18.8%), and COL11A1 (18.3%) (Figure 4A). In the SUMOPS-high subtype, the top 20 mutation frequency were detected in genes of TP53 (59.6%) TTN (55.3.1%), MUC16 (48.5%), RYR2 (43.4%), CSMD3 (42.6%), LRP1B (39.6%), ZFHX4 (38.7%), USH2A (36.6%), XIRP2 (31.1%), KRAS (30.6%), SPTA1 (28.9%), FLG (26.0%), MUC17 (24.7%), FAT3 (23.4%), ADAMTS12 (23.0%), ZNF536 (23.0%), COL11A1 (23.0%), PCDH15 (22.6%), ANK2 (22.1%), and NAV3 (22.1%), in decreasing order (Figure 4B). Although the top 20 mutated genes were common in both the SUMOPS-low and SUMOPS-high subtypes, there was a higher mutation frequency in most genes observed in the SUMOPS-high subtype than in the SUMOPS-low subtype. Furthermore, we compared the differential mutated genes between the SUMOPS-low subtype and the SUMOPS-high subtype. Figure 4C displays the top 20 mutated genes with significant mutation frequency differences between the two subtypes, including TP53, TTN, MUC16, RYR2, LRP1B, ZFHX4, XIRP2, MUC17, CDH10, RP1L1, CSMD2, PEG3, HERC2, HYDIN, SYNE1, MYH7, TRPS1, FAT1, COL22A1, and DNAH11. TP53 is known to play a crucial role in tumorigenesis with mutations associated with poor prognosis in various cancer types. The mutation status of TP53 is significantly correlated with SUMOPS suggested by the higher TP53 mutations in the SUMOPS-high subtype compared to the SUMOPS-low subtype. As high expression of immune checkpoint genes indicates a better response to immunotherapy, we examined the correlation between these mutated genes and immune checkpoint gene expression. We found that patients carrying TP53, HYDIN, or RP1L1 mutations exhibited significantly higher expression of immune checkpoint genes (CD274, PDCD1, and CTLA4) compared to their wild-type counterparts (Figure 4D–4F). This finding suggests that patients with the SUMOPS-high subtype may have a higher likelihood of responding favorably to immune checkpoint blockade therapies.

Additionally, we compared the clinical characteristics between SUMOPS-low subtype and SUMOPS-high subtypes. The results showed that the SUMOPS score was significantly lower in early-stage patients compared to late-stage patients (Figure 4G). Moreover, non-recurrent patients exhibited significantly lower SUMOPS scores compared to recurrent patients (Figure 4H).

Hayes et al. identified three subtypes of LUAD, including bronchioalveolar carcinoma (bronchioid), squamous cell carcinoma (squamoid), and large-cell carcinoma (magnoid) [27]. Among these subtypes, the bronchioid subtype has the best prognosis. We examined the correlation between SUMOPS and these three molecular subtypes using the GSE26939 cohort. The results revealed that the bronchioid subtype had the lowest SUMOPS score (Figure 4I). Meanwhile, IASLC et al. defined five new distinct histological growth patterns for conventional invasive adenocarcinomas: lepidic, acinar, papillary, micropapillary, and solid predominant adenocarcinoma [22]. These growth patterns play a significant role in the overall survival (OS) of patients, with lepidic predominant adenocarcinoma showing the best prognosis, while micropapillary and solid architectures are associated with particularly poor outcomes. Based on the analysis of the GSE58772 cohort, we further investigated the relationship between SUMOPS and the molecular subtypes (Figure 4J). The results demonstrated that the lepidic subtype had the lowest SUMOPS scores, while the solid subtype exhibited the highest SUMOPS scores. These compelling associations between SUMOPS scores and established molecular and histological subtypes provide further evidence of the prognostic value and clinical relevance of the SUMOPS model in LUAD.

CIBERSORT is a tool used to assess immune cell infiltration. We utilized CIBERSORT to evaluate the relative proportions of 22 immune cell types in all LUAD cases. The SUMOPS-high subtype exhibited higher levels of infiltration for T cell CD8, T cells CD4 memory activated, Macrophages M0, Macrophages M1, and Mast cells activated, whereas the SUMOPS-low subtype showed higher levels of infiltration for T cells CD4 memory resting, Monocytes, Macrophages M2, Dendritic cells resting, and Mast cells resting (Figure 4K).

### SUMOPS predicted the response to adjuvant chemotherapy in LUAD

**Figure 5:**
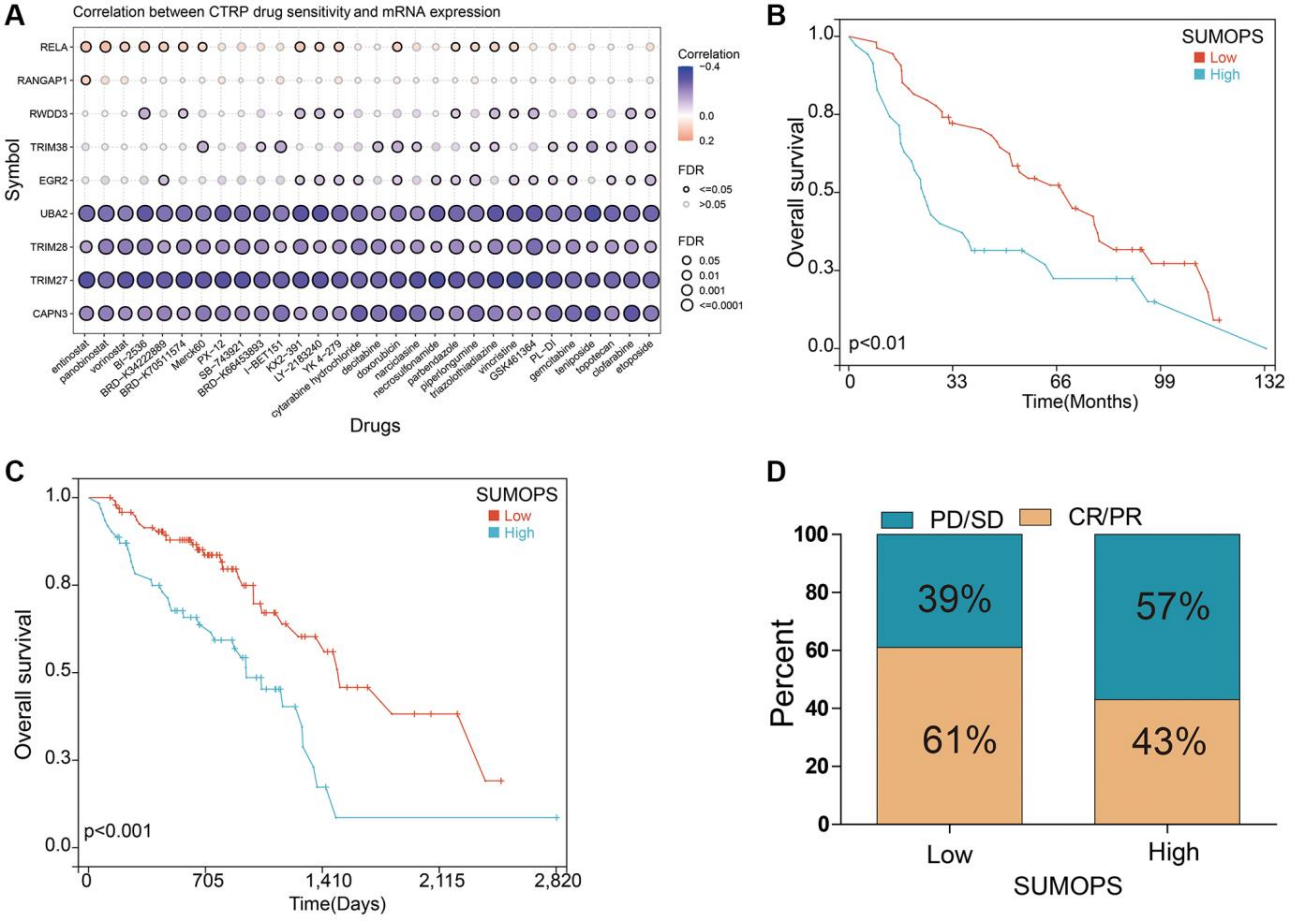
**Prediction and correlation of the sensitivity to chemotherapy drugs in LUAD.** (**A**) The correlation between GDSC drug sensitivity and SUMOPS gene expression. (**B**) The predictive value of SUMOPS in LUAD patients treated with chemotherapy in the GSE68465 cohort. (**C**) The predictive value of SUMOPS in LUAD patients treated with chemotherapy in the TCGA-LUAD cohort. (**D**) The correlation of UMOPS with response to chemotherapy in the TCGA-LUAD cohort.

Previous clinical research has consistently shown that adding adjuvant chemotherapy can significantly improve the prognosis of patients with LUAD, compared to surgical treatment alone. However, drug resistance remains a major hurdle in achieving positive treatment outcomes. To investigate potential mechanisms of drug resistance, we examined the relationship between drug sensitivity and the expression of SUMOPS genes using the Cancer Therapeutics Response Portal (CTRP) database. Our results indicated that higher gene expression levels of RELA were positively correlated with increased IC50 values of cancer therapy drugs, while UBA2, TRIM28, TRIM27, and CAPN3 showed opposite correlations (Figure 5A). These findings suggest that these genes could have important clinical implications in guiding the development of chemotherapy regimens.

Furthermore, we also analyzed the relationship between SUMOPS and the response to adjuvant chemotherapy in two cohorts that underwent chemotherapy: 85 patients from the GSE68465 and 159 patients from the TCGA. The results indicated that patients with low SUMOPS expression experienced greater benefits in terms of overall survival compared to those with high SUMOPS expression (Figure 5B, 5C). Importantly, the SUMOPS-low subtype had a higher prevalence (61% of cases) of complete or partial response (CR/PR), whereas the SUMOPS-high subtype had a higher occurrence (57%) of progressive or stable disease (PD/SD) in the TCGA-LUAD cohort (Figure 5D). In summary, SUMOPS may be a reliable tool for predicting the response of LUAD patients to adjuvant therapy. And individuals classified as the SUMOPS-low subtype may derive significant benefits from receiving adjuvant chemotherapy.

### Benefits of immune checkpoint inhibitors (ICIs) treatment in SUMOPS subgroups

**Figure 6:**
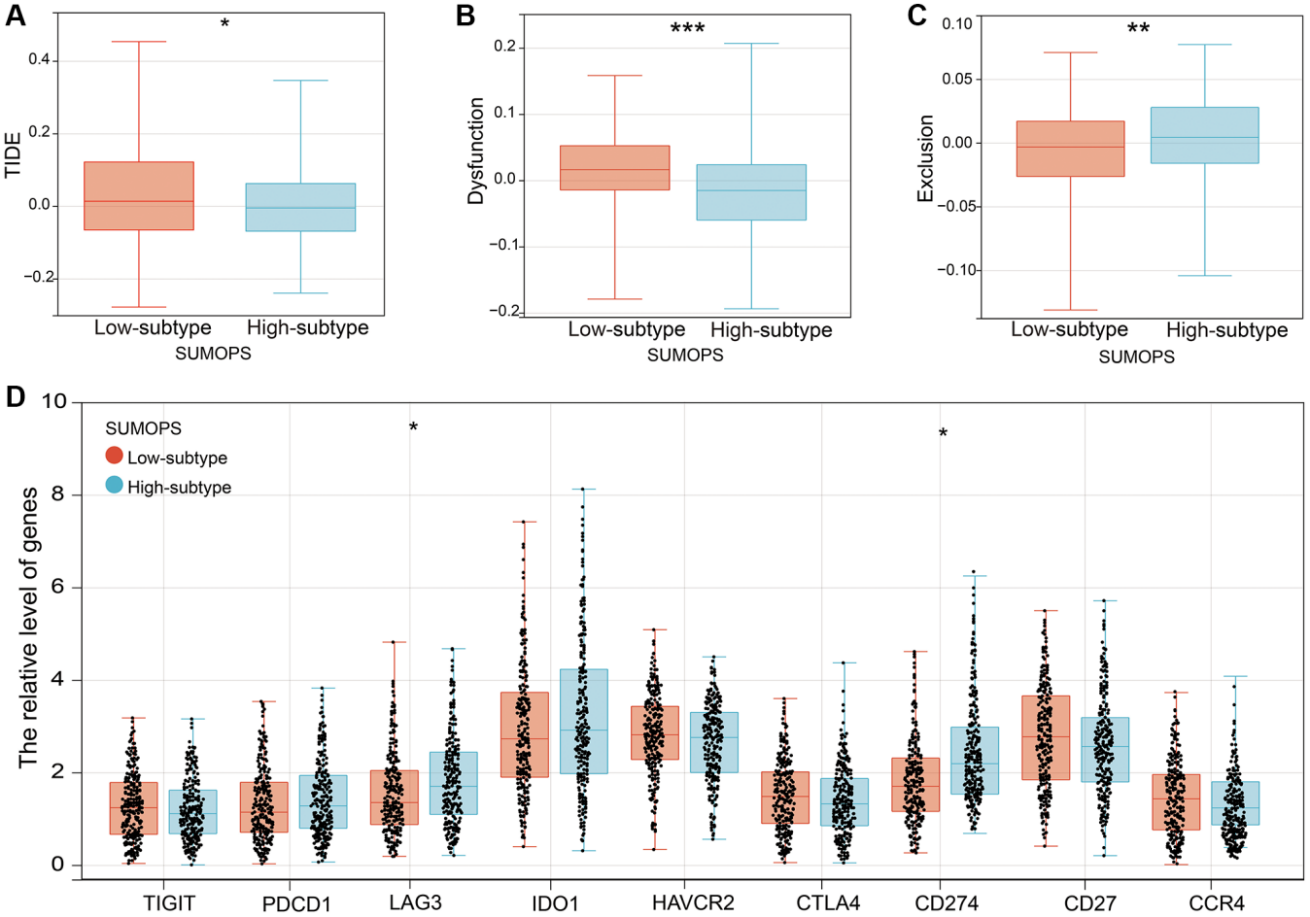
**The prognostic value of SUMOPS for ICI treatment.** (**A**–**C**) Scores of (**A**) TIDE, (**B**) T cell dysfunction and (**C**) T cell exclusion in different SUMOPS subtypes. (**D**) Box plots illustrating the relationships between SUMOPS subtypes and the expression of immune checkpoints. ^*^p < 0.05; ^**^p < 0.01, ^***^p < 0.001.

We performed the Tumor Immune Dysfunction and Exclusion (TIDE) algorithm to evaluate the potential clinical efficacy of ICIs in the SUMOPS subgroups. The lower TIDE score in the SUMOPS-high subtype suggests that patients with the SUMOPS-high subtype may derive greater benefits from ICI treatment than those with the SUMOPS-low subtype (Figure 6A). Furthermore, we investigated the relationship between the SUMOPS subgroups and T-cell exclusion and T-cell dysfunction scores. We found that the SUMOPS-low subtype exhibited higher T-cell dysfunction scores (Figure 6B), whereas the SUMOPS-high subtype demonstrated higher T-cell exclusion scores (Figure 6C).

Moreover, we examined the differential expression of immune checkpoint genes across different SUMOPS subgroups. Significantly higher expression levels of LAG3 and CD274 were observed in the SUMOPS-high subtype compared to the SUMOPS-low subtype suggesting that the SUMOPS-high subtype is more likely to benefit from immune-based therapies (Figure 6D).

### UBA2 is highly expressed in LUAD and is associated with poor prognosis in LUAD patients

**Figure 7:**
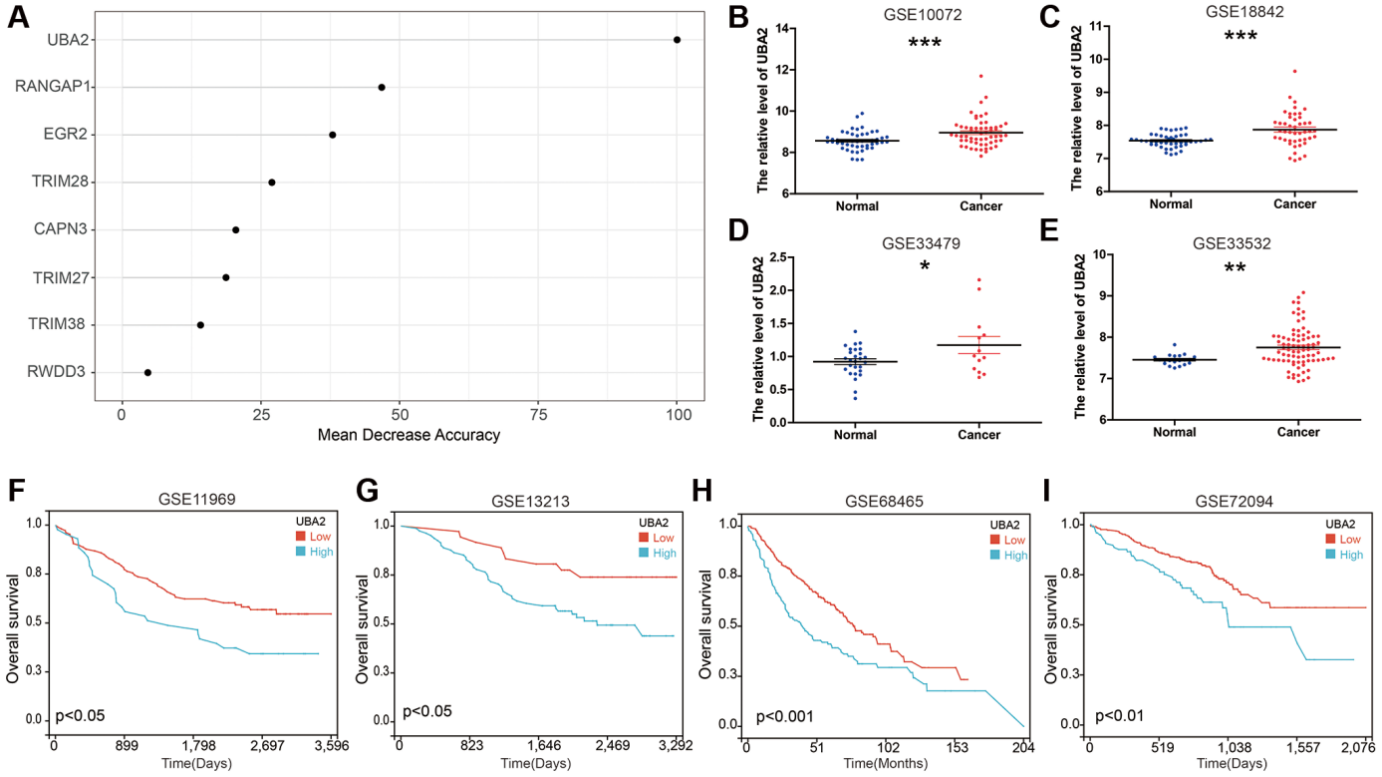
**UBA2 is highly expressed in LUAD and is associated with poor prognosis in LUAD patients.** (**A**) Random forest feature importance ranking for the SUMOPS genes; (**B**–**E**) The expression of UBA2 between LUAD and normal tissues in the (**B**) GSE10072, (**C**) GSE18842, (**D**) GSE33479 and (**E**) GSE33532 cohorts. (**F**–**I**) Prognostic analysis investigating the role of UBA2 in predicting outcomes in the (**F**) GSE11969, (**G**) GSE13213, (**H**) GSE68465 and (**I**) GSE72094 cohorts. ^*^p < 0.05; ^**^p < 0.01, ^***^p < 0.001.

We conducted forest analysis to determine the most crucial gene as a representative SUMOPS for subsequent investigation. Our analysis unveiled that UBA2 was the most significant gene among the SUMOPS genes in identifying normal tissues and LUAD tissues (Figure 7A). UBA2, alternatively referred to as SAE2, functions as a subunit within the SUMOylated E1 enzyme. UBA2 plays a critical role as one of the key enzymes responsible for regulating the levels of SUMOylation. To gain deeper insights into the molecular features of UBA2 in LUAD, we detected its transcript expression in four independent GEO cohorts (GSE10072, GSE18842, GSE33479, and GSE33532). Our results demonstrated a significant upregulation of UBA2 expression in LUAD tissues in comparison to normal tissues (Figure 7B–7E). In addition, we further validated the impact of UBA2 expression on the prognosis of LUAD patients using four independent LUAD cohorts (GSE11969, GSE13213, GSE68465, and GSE72094). The results consistently demonstrated that high expression of UBA2 is associated with poor prognosis in LUAD patients. The comprehensive analysis of these independent cohorts strengthens the significance of UBA2 as a prognostic factor in LUAD (Figure 7F–7I).

### Knockdown UBA2 inhibited the proliferation, invasion and migration of LUAD cells

**Figure 8:**
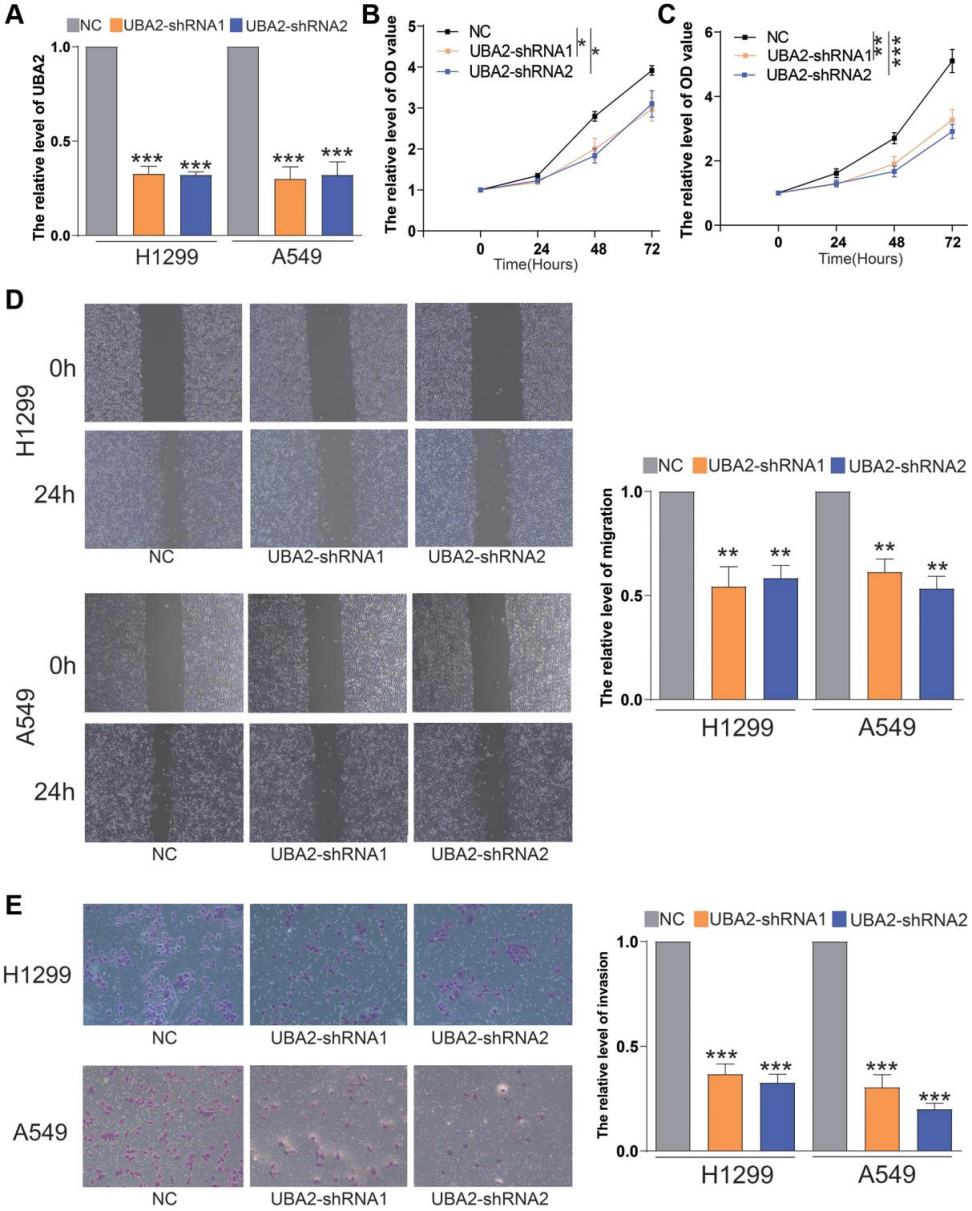
**Knockdown of UBA2 inhibited the proliferation, invasion and migration of LUAD cells.** (**A**) The UBA2 expression was measured by quantitative RT-PCR after transfecting UBA2-shRNAs in A549 and H1299. (**B**, **C**) The proliferative capacities of (**B**) H1299 and (**C**) A549 cells were measured by CCK8. (**D**) The migrative capacities of A549 and H1299 cells were measured by wound healing assay. (**E**) The invasive capacities of A549 and H1299 cells were measured by transwell assays. ^*^p < 0.05, ^**^p < 0.01 ^***^p < 0.001.

Subsequently, we performed experiments to downregulate the expression of UBA2 in A549 and H1299 cell lines and evaluated the effects of UBA2 expression alterations on the proliferation, migration, and invasion capabilities of these cells. Our results revealed that the knockdown UBA2 led to significant suppression of the proliferation, invasion, and migration capabilities of both A549 and H1299 cells (Figure 8). These imply that UBA2 may have a critical function in regulating the progression of LUAD.

## DISCUSSION

The SUMO pathway is an essential post-translational modification process that plays a crucial role in regulating protein functions [11]. The covalent attachment of SUMO proteins to target proteins, known as SUMOylation, affects various cellular processes, including transcriptional regulation, DNA repair, signal transduction, and protein stability. SUMOylation has emerged as a critical regulator in protein-protein interactions, subcellular localization, and protein stability, thereby modulating numerous cellular pathways [28]. In recent years, increasing evidence has implicated the dysregulation of the SUMOylation pathway in the development and progression of various cancers, including LUAD. Aberrant SUMOylation has been shown to impact tumor initiation, growth, metastasis, and response to therapy [29–31]. Therefore, understanding the landscape of SUMOylation pathway-related genes and their implications in LUAD can provide valuable insights into the underlying molecular mechanisms driving tumorigenesis and identify potential therapeutic targets.

### SUMOylation pathways genes predict the prognosis of patients with LUAD

In this study, we comprehensively analyzed the expression levels of 68 SUMOylation pathway-related genes in LUAD using the TCGA database. Our results revealed significant dysregulation of 53 SUMOylation pathway genes, with 38 genes showing upregulated expression and 15 genes exhibiting downregulated expression in LUAD. Furthermore, our survival analysis demonstrated that the expression levels of specific SUMOylation genes were associated with patient prognosis. Elevated expression of TRIM28, SAE1, UBA2, RELA, and SLF1 was correlated with unfavorable prognosis, while higher expression of TRIM38, CAPN3, PWDD3, TRIM27, EGR2, and RNF212B was linked to favorable prognosis in LUAD patients. These findings underscore the importance of SUMOylation pathway-related genes as potential prognostic markers and their potential as therapeutic targets.

### SUMOylation signature is associated with LUAD outcomes and tumorigenesis

To establish a robust prognostic model, we employed a LASSO-Cox regression analysis and identified nine specific genes that significantly influenced LUAD prognosis. These genes were used to construct a SUMOPS enabling accurately predicting patient outcomes. The SUMOPS score successfully distinguished between SUMOPS-low and SUMOPS-high subtypes, indicating distinct survival outcomes. Importantly, the prognostic efficacy of the SUMOPS model remained consistent across multiple independent cohorts, confirming its reliability and clinical relevance. Enrichment analysis identified significant enrichment of multiple signaling pathways in the SUMOPS-high subtype, including Glycolysis, E2F targets, Unfolded protein response, MYC targets V1, MYC targets V2, Mitotic spindle, G2M checkpoint, MTORC1 signaling, DNA repair, and PI3K/AKT/MTOR signaling. Dysregulation of these pathways is known to play a role in cancer progression.

### SUMOPS can predict LUAD subtypes

Researchers have categorized LUAD into various molecular subtypes based on whole-genome gene expression profiles. Hayes et al. classified them as bronchioid, magnoid, and squamoid subtypes [27], while the IASLC categorized them as lepidic, acinar, papillary, micropapillary, and solid predominant adenocarcinoma subtypes [22]. In our analysis, we also investigated the relationship between SUMOPS and different molecular subtypes, revealing that subtypes associated with a favorable prognosis tend to have lower SUMOPS scores.

### SUMOPS are associated with gene mutation, ICIS and TIDE

Recent research has demonstrated that lung cancer patients with TP53 mutations tend to experience worse clinical outcomes [32]. In this study, we observed a lower frequency of TP53 mutations in LUAD patients classified as the SUMOPS-low subtype, which aligns with the association between low SUMOPS scores and improved prognosis. However, some studies have also reported a positive influence of TP53 mutations on favorable responses to ICI treatments [33, 34]. Moreover, we found higher expression of CD274, PDCD1, and CTLA4 genes in LUAD patients with TP53 mutations. The complexity of immune cell infiltration within the tumor microenvironment significantly impacts the effectiveness of LUAD treatment. We further observed a higher infiltration of M2 macrophages and a lower infiltration of M1 macrophages in the SUMOPS-low subtype. Macrophages can exhibit two primary phenotypes: pro-inflammatory M1 macrophages and pro-tumor M2 macrophages. However, a study by Mehrdad et al. suggested that the infiltration of M2 macrophages improves the prognosis of non-small cell lung cancer (NSCLC) patients [35]. Another study focusing on the immune microenvironment in LUAD found enrichment of M2 macrophages in patients with extended survival and lower mutation burden [36]. These results suggest that LUAD patients with SUMOPS-high subtype may respond better to immunotherapy.

The TIDE algorithm has been developed to simulate two major immune evasion mechanisms in tumors, aiming to predict the response to ICI therapy [26, 37, 38]. The TIDE prediction score is positively correlated with the likelihood of tumor immune evasion. This suggests that patients with higher TIDE scores are less likely to benefit from ICI treatment. In our study, we found that the SUMOPS-high subtype exhibited lower TIDE scores and higher T-cell exclusion scores, whereas the SUMOPS-low subtype had higher TIDE scores and T-cell dysfunction scores. As a result, patients within the SUMOPS-low subtype may experience immune evasion due to T-cell dysfunction, leading to poorer responses to ICIs. Conversely, high-risk patients within the SUMOPS-high subtype may potentially benefit more from ICI treatment.

Blocking immune checkpoints can reduce tumor cell immune evasion and activate immune responses within the tumor microenvironment [39, 40]. The significantly higher expression of immune checkpoint genes including LAG3 and CD274 in the SUMOPS-high subtype compared to the SUMOPS-low subtype further supports the notion that patients within the SUMOPS-high subtype may be more suitable for immune-based therapies.

### SUMOPS predicts responses to adjuvant chemotherapy

Among LUAD patients receiving adjuvant chemotherapy, those classified as the SUMOPS-low subtype showed improved overall survival, and a higher rate of complete or partial responses compared to patients in the SUMOPS-high subtype. These findings suggest that the SUMOPS model could serve as a valuable tool for predicting the response to adjuvant chemotherapy in LUAD patients and guiding personalized immunotherapy strategies.

### SUMOylation presentative gene UBA2 effects LUAD proliferation

Finally, random forest analysis highlighted the crucial role of UBA2 in accurately distinguishing LUAD. Therefore, we focused on the role of UBA2 in lung adenocarcinoma (LUAD) and its association with patient prognosis. Our results demonstrated that UBA2 was highly expressed in LUAD tissues and was significantly associated with poor prognosis in LUAD patients. The downregulation of UBA2 significantly inhibited the proliferation, invasion, and migration capabilities of these cells, indicating that UBA2 may play a critical role in the regulation of LUAD progression. It highlights UBA2 as a potential therapeutic target for intervention strategies aimed at inhibiting tumor growth and metastasis in LUAD. It is important to note that further studies are needed to elucidate the specific molecular mechanisms through which UBA2 contributes to the progression of LUAD. And future investigations should explore the potential of targeting UBA2 as a therapeutic strategy for LUAD treatment.

In conclusion, our study provides novel evidence implicating dysregulation of the SUMOylation pathway and prognostic value of the SUMOPS score in LUAD progression and response to treatment. However, several limitations must be considered. As a retrospective analysis, our study is inherently limited by potential biases in the publicly available TCGA dataset, which may not fully represent the diversity of clinical presentations. Prospective clinical validation is still needed to rigorously determine the practical utility and predictive performance of the SUMOPS model. Additionally, while our findings point to the significance of SUMO pathway alterations, more comprehensive investigation is required to fully elucidate its complex interplay with other molecular changes influencing LUAD tumor biology and patient outcomes. Addressing these limitations through well-designed prospective studies and more in-depth molecular characterization would help translate our results toward clinical utility. Nevertheless, this study provides a foundation for further exploring the prognostic and therapeutic implications of the SUMOylation pathway in LUAD.

## CONCLUSION

In summary, this study provides a comprehensive overview of the SUMOylation pathway-related genes in LUAD and their potential impact on patient prognosis and treatment responses. It also highlights the value of the SUMOPS in estimating prognosis, guiding adjuvant chemotherapy, and predicting immunotherapy efficacy. The proposed SUMOPS scoring model may contribute to the prognostic assessment and improvement of treatment outcomes for LUAD patients.

## Supplementary Materials

Supplementary Figure 1
