# Supplementary material for: The prognosis, chemotherapy and immunotherapy efficacy of the SUMOylation pathway signature and the role of UBA2 in lung adenocarcinoma
Source: Aging (Albany NY). 2024 Feb 23;16(5):4378–95. doi: 10.18632/aging.205594 (PMC10968705; doi:10.18632/aging.205594)
Supplement: Supplementary Figure 1 [file aging-16-205594-s001.pdf]

SUPPLEMENTARY FIGURE

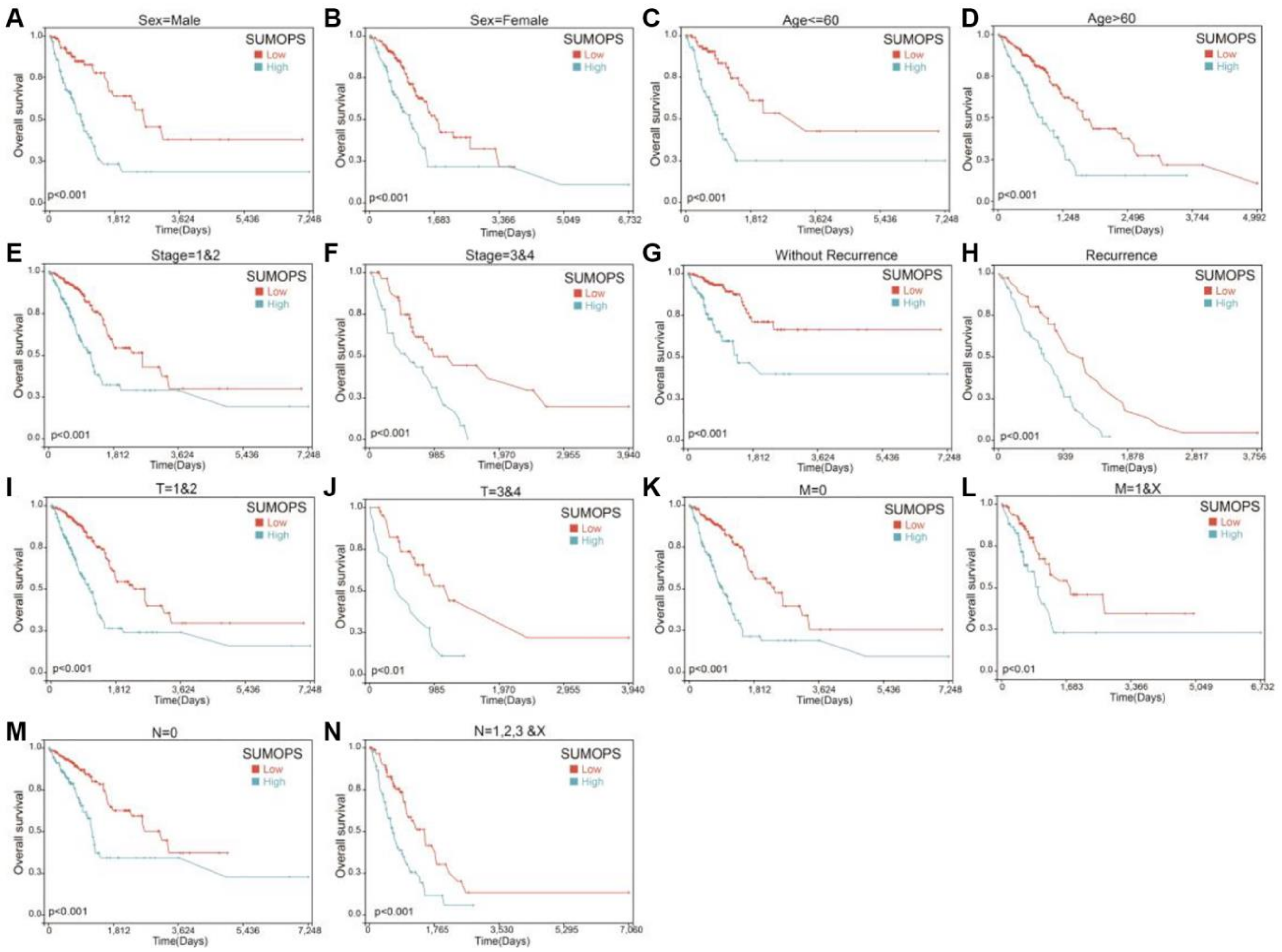

Supplementary Figure 1. The prognostic value of the SUMOPS for different clinical factors in LUAD. Comparisons of the overall survival differences between the SUMOPS-low and the SUMOPS-high subtypes in the LUAD patient subgroups categorized by (**A**) male and (**B**) female, (**C**) age ≤60 years, (**D**) age >60 years, (**E**) stage = 1–2, (**F**) stage = 3–4, (**G**) without recurrence, (**H**) recurrence, (**I**) stage T = 1–2, (**J**) stage T = 3–4, (**K**) stage M = 0, (**L**) stage M = 1&X, (**M**) stage N = 0, and (**N**) stage N = 1,2,3&X.
